# Oridonin exerts anticancer effect on osteosarcoma by activating PPAR-γ and inhibiting Nrf2 pathway

**DOI:** 10.1038/s41419-017-0031-6

**Published:** 2018-01-11

**Authors:** Ying Lu, Yang Sun, Jianwei Zhu, Lisha Yu, Xiubo Jiang, Jie Zhang, Xiaochen Dong, Bo Ma, Qi Zhang

**Affiliations:** 1 School of Pharmaceutical Sciences, Nanjing Tech University (NanjingTech), 30 South Puzhu Road, Nanjing, 211816 The People’s Republic of China; 2 Key Laboratory of Flexible Electronics (KLOFE) & Institute of Advanced Materials (IAM), Jiangsu National Synergetic Innovation Center for Advanced Materials (SICAM), Nanjing Tech University (NanjingTech), 30 South Puzhu Road, Nanjing, 211816 The People’s Republic of China

## Abstract

Osteosarcoma is the most common high-grade human primary malignant bone sarcoma with lower survival in the past decades. Oridonin, a bioactive diterpenoid isolated from *Rabdosia rubescens*, has been proved to possess potent anti-cancer effects. However, its potential mechanism still remains not fully clear nowadays. In this study, we investigated the anticancer effect of oridonin on human osteosarcoma and illuminated the underlying mechanisms. In vitro, oridonin inhibited the cell viability of various osteosarcoma cells. We demonstrated that oridonin induced mitochondrial-mediated apoptosis by increasing Bax/Bcl-2 ratio, loss of mitochondrial membrane potential (MMP), triggering reactive oxygen species (ROS) generation and activating caspase-3 and caspase-9 cleavage in MG-63 and HOS cells. Moreover, we found that oridonin triggered ROS by inhibiting NF-E2-related factor 2 (Nrf2) pathway and induced mitochondrial apoptosis via inhibiting nuclear factor-κB (NF-κB) activation by activating Peroxisome Proliferator-Activated Receptor γ (PPAR-γ) in MG-63 and HOS cells. We further confirmed the results by PPAR-γ inhibitor GW9662, PPAR-γ siRNA as well as overexpression of PPAR-γ and Nrf2 in vitro. Furthermore, our in vivo study showed that oridonin inhibited tumor growth with high safety via inducing apoptosis through activating PPAR-γ and inhibiting Nrf2 activation in xenograft model inoculated HOS tumor. Taken together, oridonin exerted a dramatic pro-apoptotic effect by activating PPAR-γ and inhibiting Nrf2 pathway in vitro and in vivo. Therefore, oridonin may be a promising and effective agent for human osteosarcoma in the future clinical applications.

## Introduction

Osteosarcoma is the most common high-grade primary malignant bone tumor and prevailing in children and young adults^1^. The standard therapy for osteosarcoma consists of neoadjuvant chemotherapy, surgical excision and chemotherapy again after resection^2^. Despite the advances in surgical technique, relapse and chemotherapy resistance is still the main challenges for physicians^3^. Over the last three decades, the 5-year survival of patients with local osteosarcoma has improved to 60%, but has remained essentially unchanged^4^. Consequently, it is indispensable to develop more effective therapies against osteosarcoma.

Peroxisome Proliferator-Activated Receptor γ (PPAR-γ), one kind of the nuclear hormone receptor family, plays a key role in carcinogenesis^5^. PPAR-γ has been considered as a tumor suppressor in many solid cancers including human breast, prostate, colon, and lung cancer^6^. PPAR-γ activation possesses potential anticancer effects both in solid cancer and in leukemia through induction of apoptosis, inhibition of cell proliferation, terminal differentiation^6^. Increasing evidence has demonstrated that PPAR-γ activation may be considered as a possible intervention in osteosarcoma^7–11^. PPAR-γ agonists can promote osteoblastic differentiation of osteosarcoma cells^12^ and suppress its proliferation^13^. In addition, activating PPAR-γ exerts the anticancer effects through the inhibition of the activation of nuclear factor-κB (NF-κB)^14,15^. NF-κB family is consisted of five related transcription factors regulating gene transcription in various physiological conditions^16^. It is well known that aberrant NF-κB activity is invoved in the cell proliferation, the subversion of apoptosis and cancer development^17^. Overactive NF-κB signaling pathway regulates many carcinogenic genes^16^ and is reported in numerous cancers such as leukemias, breast cancer, prostate cancer, melanoma, lymphomas^18^, especially in osteosarcoma^19^. Notably, a recent study further demonstrated the activation of NF-κB leads to increase cell proliferation in osteosarcoma and hinder osteoblastic differentiation^16^. Therefore, blockage of NF-κB activation sensitizes osteosarcoma to chemotherapeutic agents^19^.

NF-E2-related factor 2 (Nrf2), a transcription factor that modulates the level of reactive oxygen species (ROS), detoxifying agents and antioxidants^20^. Currently, substantial evidence has suggested that Nrf2 pathway is activated to trigger the expression of antioxidant response element (ARE) target genes, including NAD(P)H:quinone oxidoreductase 1 (NQO1) and heme oxygenase-1 (HO-1), which attenuate cellular oxidative stress^21,22^. It has been clearly documented that Nrf2 plays a crucial role in the progression, invasion, and metastasis of various cancers^23^. The elevated expression of Nrf2 has been found in head and neck^24^, gall bladder^25^ and lung cancer^26^. Documents showed that the accumulation of Nrf2 in the nuclei is closely related to bone metastasis of patients with osteosarcoma, indicating that activation of Nrf2 is vital for the development and progression of osteosarcoma^27^. However, the significance of Nrf2 in osteosarcoma tissue need to be further studied.

Oridonin, a diterpenoid isolated from medicinal herb *Rabdosia rubescens*, has been proved to possess potent antitumor effect on various cancers, such as colon cancer cells^28^, lymphoma cells^29^, breast cancer cells^30^ and leukemia cells^31^. It has been documented that oridonin may induce apoptosis in numerous cancers^32,33^. In addition, JDA-202 and adenanthin, which possess the same active site structure with oridonin, has been proved to target peroxiredoxins to exert their antitumor activity^34,35^. Moreover, there is an enormous work investigating the mechanism of the antitumor effect of oridonin^36^. However, its potential molecular mechanism of the antitumor effect is still not fully clear, especially on osteosarcoma. Therefore, we explored the anticancer effect of oridonin on human osteosarcoma and the underlying mechanisms. We demonstrated that oridonin exerted a dramatic pro-apoptotic effect by activating PPAR-γ and inhibiting Nrf2 pathway in vitro and in vivo.

## Results

### Oridonin induced apoptosis in human osteosarcoma cells

**Fig. 1:**
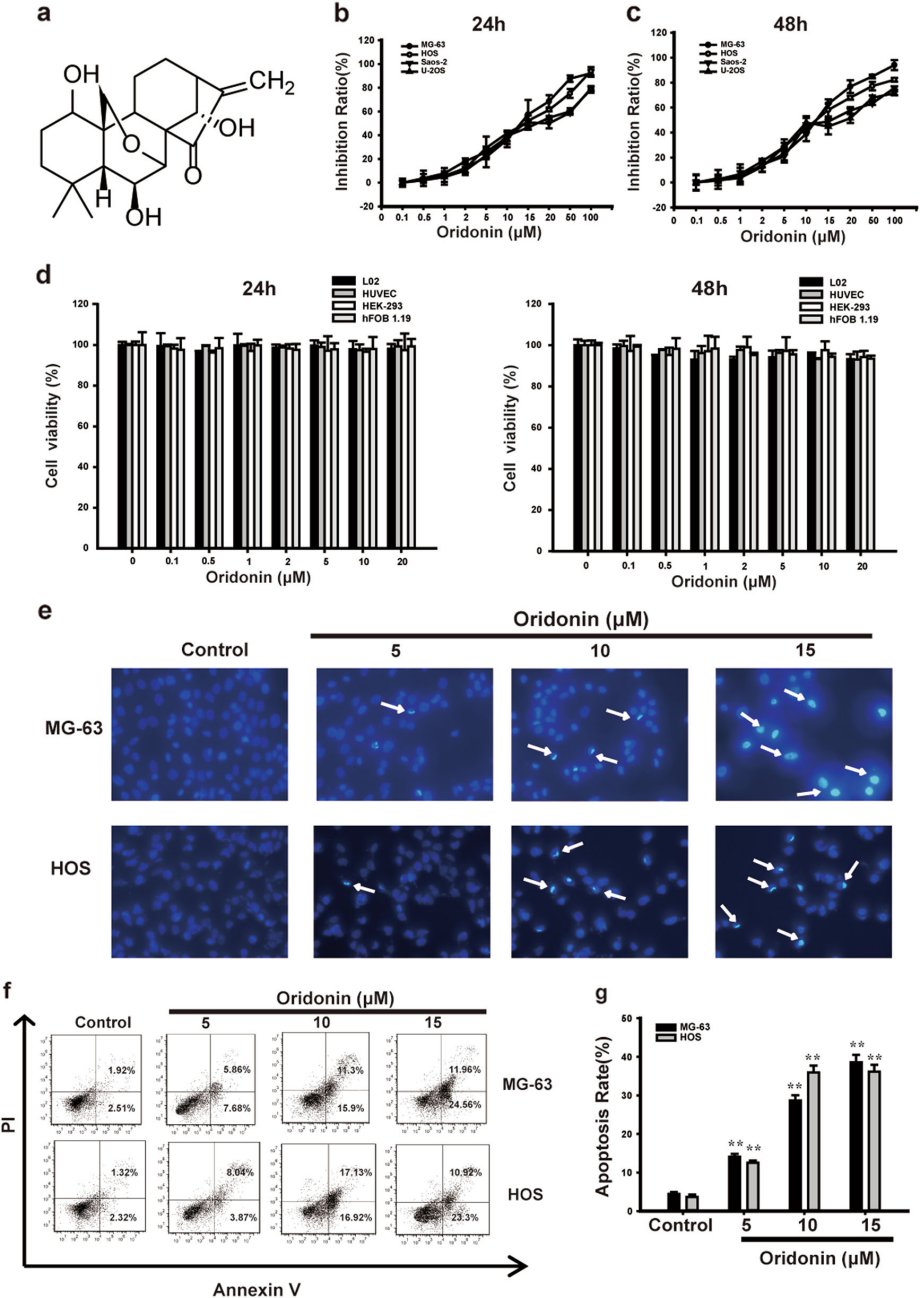
Oridonin induced apoptosis in human osteosarcoma cells. **a** Structure of oridonin (C_20_H_28_O_6_, MW = 364.4327). **b** The effect of oridonin on the cell viability of MG-63, HOS, Saos-2 and U-2OS cells after 24 h treatment. **c** The effect of oridonin on the cell viability of MG-63, HOS, Saos-2, and U-2OS cells after 48 h treatment. **d** The effect of oridonin on the cell viability of human normal cells L02, HUVEC, HEK-293 and human normal osteogenic cell lines hFOB 1.19 after 24 h treatment. **e** After given oridonin (5, 10, and 15 µM) for 24 h, the nuclear morphologic changes of MG-63 and HOS cells were observed by fluorescent microscope (400×). Apoptosis bodies and nuclei pyknosis were observed in apoptosis cells. **f**, **g** The apoptotic rates of cells induced by oridonin in MG-63 and HOS cells detected by Annexin V/PI double-staining assay. The results are representative of at least three independent experiments and shown as mean ± SD. **P < 0.01 compared with control group

The chemical structure of oridonin was shown in Fig. 1a. To evaluate the inhibitory effect of oridonin on cell viability of human osteosarcoma cell lines including MG-63, HOS, Saos-2 and U-2OS cells, we performed MTT test at different concentrations after 24 or 48 h treatment of oridonin. After 24 h of treatment, the IC50 (the concentration of drug inhibiting 50% of cells) values of MG-63, HOS, Saos-2 and U-2OS cells were 12.29 ± 1.02, 13.22 ± 0.86, 19.72 ± 0.75, 19.56 ± 0.47 μM, respectively (Fig. 1b). After 48 h of treatment, the IC50 values of MG-63, HOS, Saos-2, and U-2OS cells were 10.88 ± 0.72, 11.91 ± 0.58, 17.32 ± 0.42, 17.71 ± 0.62 μM, respectively (Fig. 1c). MG-63 and HOS cell lines were more susceptive to oridonin, thus we used these two cell lines with 5, 10, and 15 μM of oridonin treatment for 24 h in the following experiments. We used several human cancer cell lines to compare the activity of oridonin to osteosarcoma. MTT assays showed that after oridonin treatment for 24 h, the IC50 (the concentration of drug inhibiting 50% of cells) values of A549, BXPC-3, HCT-116, Hela, SKOV-3, AGS, K562 cells were 56.47 ± 0.17, 63.91 ± 0.11, 19.18 ± 0.27, 65.5 ± 0.16, 23.56 ± 0.27, 23.36 ± 0.24, 21.78 ± 0.25 μM, respectively (Supplementary Figs. 1a, b). In addition, we chose cisplatin as the positive compound to compare the inhibitory effect of oridonin on osteosarcoma. We found that after treatment of cisplatin for 24 h, the IC50 values of MG-63, HOS, Saos-2 and U-2OS cells were 24.26 ± 0.29, 30.81 ± 0.21, 30.02 ± 0.22, 26.61 ± 0.25 μM respectively. After 48 h of treatment, the IC50 values of MG-63, HOS, Saos-2 and U-2OS cells were 21.1 ± 0.28, 29.52 ± 0.22, 27.6 ± 0.26, 23.82 ± 0.24 μM respectively (Supplementary Figs. 2a, b). These results showed that osteosarcoma was more susceptive to oridonin. Moreover, we used human normal cells L02, HUVEC, HEK-293 and human normal osteogenic cell lines hFOB 1.19 to evaluate the safety of the concentrations of oridonin we chose in vitro. MTT assays showed that there were no significant effects on cell viability of L02, HUVEC, HEK-293 and hFOB 1.19 cells after treatment with the same concentration of oridonin (Fig. 1d). These data suggested that oridonin had a selectively inhibitory effect on osteosarcoma cells but not on normal cells.

We used DAPI staining assay to test if oridonin induced apoptosis in MG-63 and HOS cells. The result showed that the control cells were in round shapes, whereas cells treated with oridonin had apoptotic chromatin condensation and DNA fragmentation in a dose-dependent manner (Fig. 1e). We confirmed the pro-apoptotic effect of oridonin by Annexin V/PI staining assay. As shown in Figs. 1f, g, the number of apoptotic cells was remarkably increased by oridonin in a dose-dependent manner compared with control group. The quantitative analysis for the percentage of apoptotic cells showed that oridonin significantly induced apoptosis in MG-63 and HOS cells. Our findings demonstrated that oridonin induced typical apoptosis in human osteosarcoma cells.

### Oridonin induced mitochondrial apoptosis in MG-63 and HOS cells

**Fig. 2:**
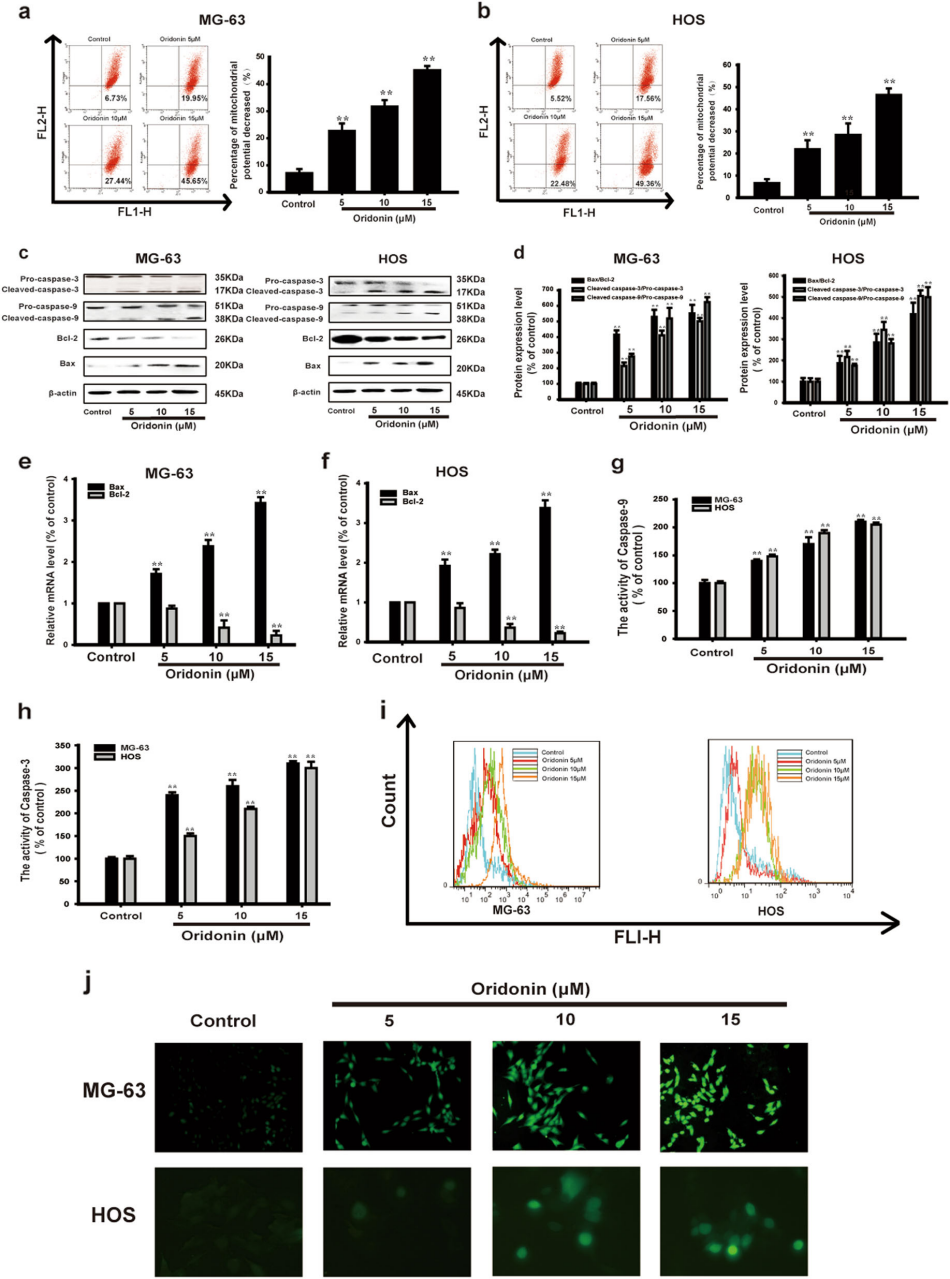
Oridonin induced mitochondrial apoptosis in MG-63 and HOS cells. **a**, **b** The change of ΔΨ in MG-63 and HOS cells was monitored by JC-1 staining and analyzed by flow cytometry. The results are representative of at least three independent experiments and shown as mean ± SD. **P < 0.01 compared with control group. **c** The protein expression of Pro-caspase-3, Cleaved caspase-3, Pro-caspase-9 and Cleaved caspase-9, Bcl-2, Bax were detected by Western blot analysis. β-actin was used as the loading control. **d** Gray scale analysis was performed to determine the relative ratios of Bax/Bcl-2, Cleaved caspase-3/Pro-caspase-3 and Cleaved caspase-9/Pro-caspase-9. The results are shown as means ± SD from three independent experiments. **P < 0.01 compared with control group. **e**, **f** The mRNA levels of Bax and Bcl-2 in MG-63 and HOS cells were measured by real-time PCR. The results are representative of at least three independent experiments and shown as mean ± SD. **P < 0.01 compared with control group. **g**, **h** The activities of Caspase-9 and Caspase-3 of MG-63 and HOS cells. The results are representative of at least three independent experiments and shown as mean ± SD. **P < 0.01 compared with control group. **i** The level of intracellular ROS was measured by flow cytometry. **j** The intracellular ROS generation in MG-63 and HOS cells was detected by Fluorescence microscopy. The results are representative of at least three independent experiments and shown as mean ± SD. **P < 0.01 compared with control group

To illustrate the mechanism of oridonin-induced apoptosis, mitochondrial membrane potential (MMP) was detected by flow cytometry. We found that MMP sharply decreased following oridonin treatment in a dose-dependent manner (Figs. 2a, b). Moreover, we detected the levels of the apoptosis related proteins such as Bcl-2, Bax, Cleaved caspase-9, Cleaved caspase-3 by Western Blot. After treatment with oridonin for 24 h, the pro-apoptotic protein Bax expression was increased while the anti-apoptotic protein Bcl-2 expression was decreased in a dose-dependent manner (Fig. 2c). The ratio of Bax/Bcl-2 was dose-dependently increased by oridonin (Fig. 2d). The mRNA levels of Bcl-2 and Bax was also regulated by oridonin accordingly (Figs. 2e, f). Besides, Caspase-3 and Caspase-9 cleavage were remarkably activated after oridonin treatment (Fig. 2c). The ratios of Cleaved caspase-9/Pro-caspase-9 and Cleaved caspase-3/Pro-caspase-3 were significantly increased after oridonin treatment (Fig. 2d). We next determined the activities of Caspase-9 and Caspase-3 in MG-63 and HOS cells after oridonin treatment. We found that oridonin increased the activities of Caspase-9 and Caspase-3 (Figs. 2g, h).

The oxidative stress is an important factor leading to mitochondrial dysfunction, thus causes excessive generation of ROS and the imbalance of oxidation system and antioxidation system^37^. Therefore, we examined the effect of oridonin on ROS generation in MG-63 and HOS cells. Our results showed that oridonin increased the generation of ROS in MG-63 and HOS cells (Figs. 2i, j). The results demonstrated that oridonin induced apoptosis by triggering excessive generation of ROS. Taken together, all these findings indicated that oridonin induced mitochondrial apoptosis in MG-63 and HOS cells.

### Oridonin inhibited NF-κB signaling pathway via activating PPAR-γ in vitro

**Fig. 3:**
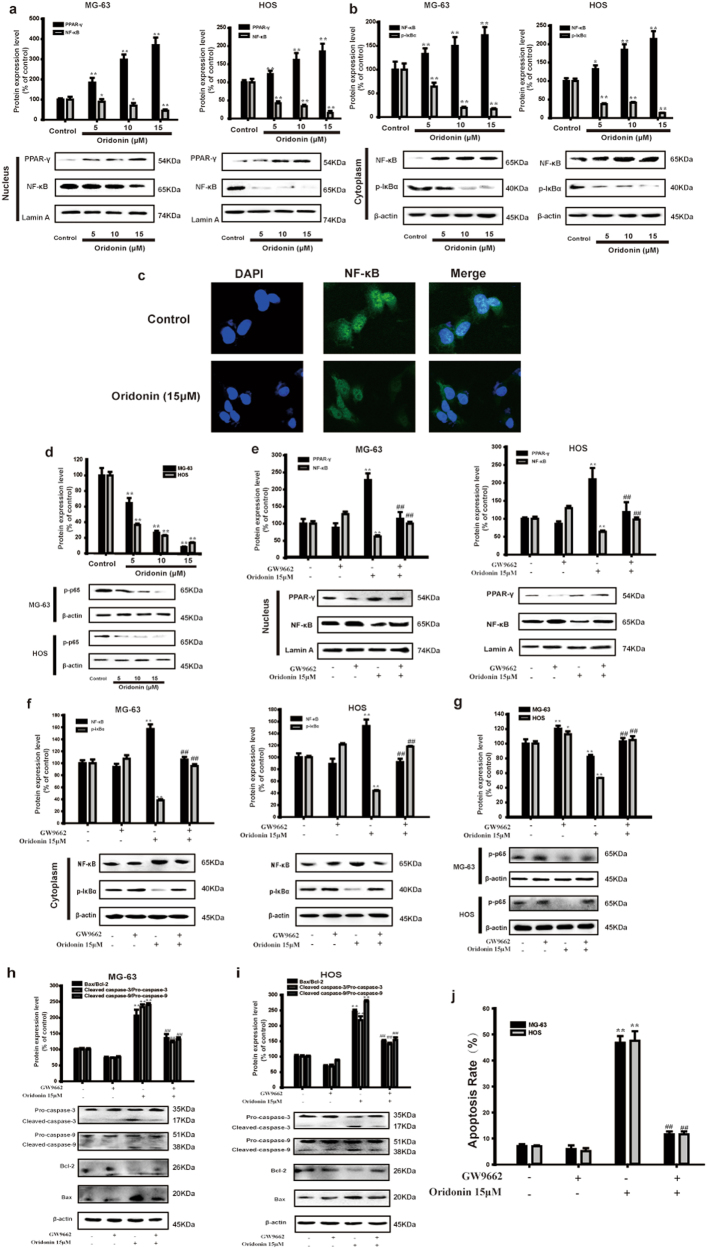
Oridonin inhibited NF-κB signaling pathway via activating PPAR-γ in vitro. **a**-**d** MG-63 and HOS cells were treated with oridonin (5, 10 and 15 µM) for 24 h. **a** Nuclear protein expression of PPAR-γ and NF-κB and **b** Cytoplasmic protein expression of NF-κB and p-IκBα were detected by Western blot. Lamin A and β-actin were used as nuclear and cytoplasmic markers, respectively. Gray scale analysis was performed to determine the relative ratios of PPAR-γ, NF-κB and p-IκBα. The results are shown as means ± SD from three independent experiments. *P < 0.05, **P < 0.01 compared with control group. **c** Immunofluorescence was performed to analyze NF-κB p65 nuclear translocation in MG-63 cells. **d** The protein expression of p-p65 in MG-63 and HOS cells were determined by Western blot. β-actin was used as an internal control. Gray scale analysis was performed to determine the relative ratio of p-p65. The results are shown as means ± SD from three independent experiments. **P < 0.01 compared with control group. **e**-**g** MG-63 and HOS cells were incubated with oridonin (15 µM) for 24 h with pretreatment of GW9662. **e** Nuclear protein expression of PPAR-γ and NF-κB and **f** Cytoplasmic protein expression of NF-κB and p-IκBα were detected by Western blot. Lamin A and β-actin were used as nuclear and cytoplasmic markers, respectively. Gray scale was performed to determine the relative ratios of PPAR-γ, NF-κB and p-IκBα. The results are shown as means ± SD from three independent experiments. **P < 0.01 compared with control group, ^##^*P* < 0.01 compared with 15 µM oridonin group. **g** The protein expression of p-p65 in MG-63 and HOS cells were determined by Western blot. β-actin was used as an internal control. Gray scale analysis was performed to determine the relative ratio of p-p65. The results are shown as means ± SD from three independent experiments. *P < 0.05, **P < 0.01 compared with control group; ^*##*^*P* < 0.01 compared with 15 µM oridonin group. **h**, **i** The protein expression of Pro-caspase-3, Cleaved caspase-3, Pro-caspase-9 and Cleaved caspase-9, Bcl-2, Bax in MG-63 and HOS cells were detected by Western blot analysis. Gray scale analysis was performed to determine the relative ratios of Bax/Bcl-2, Cleaved caspase-3/Pro-caspase-3 and Cleaved caspase-9/Pro-caspase-9. The results are shown as means ± SD from three independent experiments. **P < 0.01 compared with control group; ^##^*P* < 0.01 compared with 15 µM oridonin group. **j** The respective apoptosis rates of different groups were measured by Annexin V/PI staining. The results are representative of at least three independent experiments and shown as mean ± SD. **P < 0.01 compared with control group; ^##^*P* < 0.01 compared with 15 µM oridonin group

NF-κB signaling pathway is extremely activated in human osteosarcoma^17^. The activation of PPAR-γ exerts the anticancer effects through inhibition of NF-κB^14,15^. To further illuminate the detailed mechanisms, we examined whether oridonin could affect PPAR-γ expression and NF-κB activation. As shown in Figs. 3a, b, oridonin significantly increased the protein expression of PPAR-γ in MG-63 and HOS cells in a dose-depended manner. The levels of NF-κB in cytoplasm and nucleus were monitored to validate whether oridonin could inhibit NF-κB activation in MG-63 and HOS cells. Oridonin significantly increased NF-κB in the cytoplasm and decreased NF-κB inside the nucleus (Figs. 3a, b). The inhibitory effect of oridonin on nuclear translocation of NF-κB was confirmed by immunofluorescence staining (Fig. 3c). Oridonin also inhibited the protein expression of phosphorylation of NF-κB p65 (Fig. 3d).

To determine whether oridonin inhibited NF-κB activation by activating PPAR-γ, MG-63 and HOS cells were pretreated with GW9662, a well-known inhibitor of PPAR-γ. The inhibitory effect of oridonin on activation and nuclear translocation of NF-κB was reversed by the presence of GW9662 (Figs. 3e-g). These findings supported the fact that oridonin inhibited activation of NF-κB by activating PPAR-γ in MG-63 and HOS cells. We further detected the effects of oridonin on the protein expression of Bax, Bcl-2, Cleaved caspase-9 and Cleaved caspase-3 in the presence of GW9662. After pretreatment with GW9662, the effects of oridonin on the protein expression of apoptosis related proteins was obviously reversed (Figs. 3h, i). The flow cytometry analysis demonstrated that the percentage of early and late apoptotic cells of oridonin group was withdrawn by oridonin (Fig. 3j). Moreover, we silenced PPAR-γ with PPAR-γ siRNA in MG-63 and HOS cells. Remarkably, the inhibitory effect of oridonin on the nuclear translocation and activation of NF-κB was reversed by PPAR-γ siRNA transfection (Supplementary Fig. 3a-c). Furthermore, we overexpressed PPAR-γ by PPAR-γ plasmid in MG-63 and HOS cells. Both the presence of oridonin and PPAR-γ plasmid inhibited the nuclear translocation and activation of NF-κB (Supplementary Fig. 3d-f). These findings suggested that oridonin inhibited activation of NF-κB by activating PPAR-γ in MG-63 and HOS cells.

### Oridonin induced apoptosis by triggering ROS generation via inhibiting Nrf2 signaling pathway in vitro

**Fig. 4:**
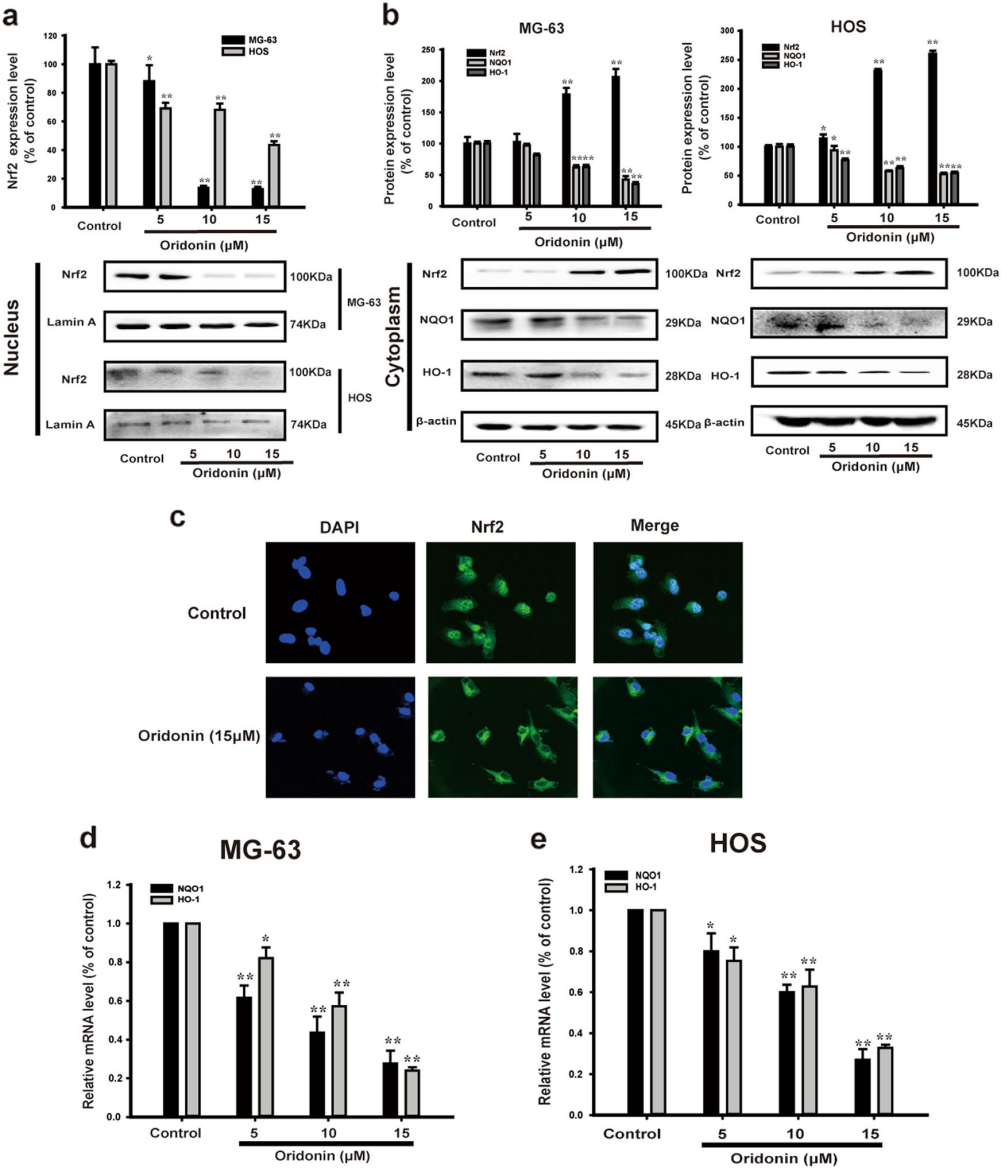
Oridonin inhibited Nrf2 signaling pathway in vitro. **a** The nuclear protein expression of Nrf2 in MG-63 and HOS cells were determined by Western blot analysis. Lamin A was used as the loading control. Gray scale analysis was performed to determine the relative ratio of Nrf2. The results are shown as means ± SD from three independent experiments. *P < 0.05, **P < 0.01 compared with control group. **b** The cytoplasmic protein expression of Nrf2, NQO1, HO-1 in MG-63 and HOS cells were determined by Western blot analysis. β-actin was used as the loading control. Gray scale analysis was performed to determine the relative ratios of Nrf2, NQO1 and HO-1. The results are shown as means ± SD from three independent experiments. *P < 0.05, **P < 0.01 compared with control group. **c** Immunofluorescence was performed to analyze Nrf2 nuclear translocation in MG-63 cells. **d**, **e** The mRNA levels of NQO1, HO-1 in MG-63 and HOS cells were measured by real-time PCR. The results are representative of at least three independent experiments and shown as mean ± SD. *P < 0.05, **P < 0.01 compared with control group

ROS is strictly controlled by an inducible antioxidant program primarily regulated by the transcription factor Nrf2^38^. The evidence has documented that the protein expression of Nrf2 was significantly lower in normal peritumor tissue than in osteosarcoma tissue^27^. To determine whether the generation of ROS triggered by oridonin was correlated with inhibiting Nrf2 activation, we examined the nuclear translocation of Nrf2 in MG-63 and HOS cells. Western blot analysis showed that oridonin treatment significantly inhibited the nuclear expression of Nrf2 and raised the cytoplasmic expression of Nrf2 compared with those in the control group (Figs. 4a, b). Oridonin also reversed the nuclear translocation of Nrf2 (Fig. 4c). The downstream of Nrf2 signaling pathway in mammals include NQO1 and HO-1^20^. Quantitative real-time PCR analysis revealed that NQO1 and HO-1 were downregulated by oridonin in the osteosarcoma cells (Figs. 4d, e). As Fig. 4b shown, oridonin suppressed the protein expression of NQO1 and HO-1 as well.

**Fig. 5:**
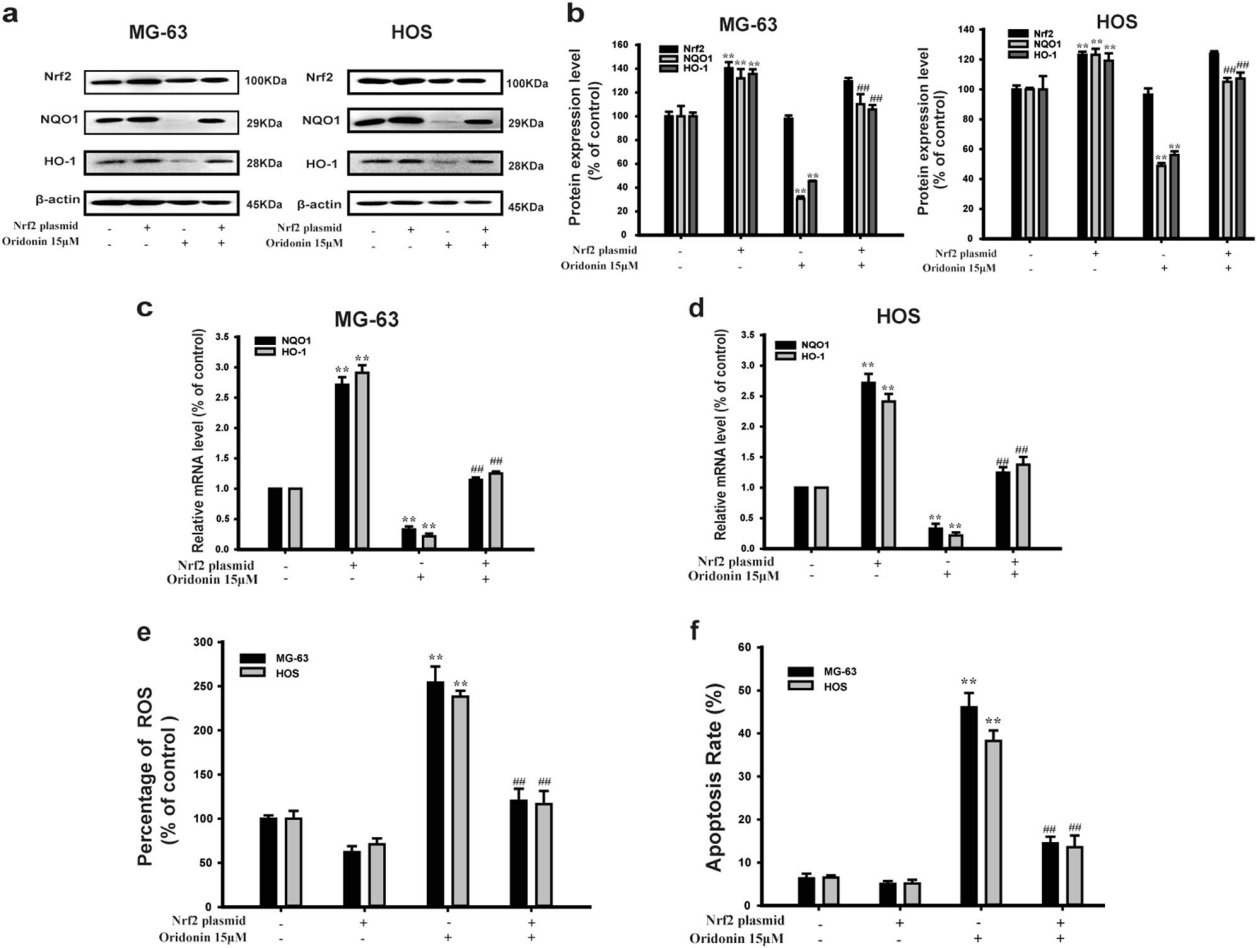
Oridonin induced apoptosis by triggering ROS generation via inhibiting Nrf2 signaling pathway in vitro. **a**-**e** MG-63 and HOS cells were transfected with Nrf2 plamid for 8 h followed by with/without 15 µM oridonin for 24 h. **a** The protein expression of Nrf2, NQO1, HO-1 in MG-63 and HOS cells were determined by Western blot analysis. β-actin was used as the loading control. **b** Gray scale analysis was performed to determine the relative ratios of Nrf2, NQO1, HO-1. The results are shown as means ± SD from three independent experiments. **P < 0.01 compared with control group; ^##^*P* < 0.01 compared with 15 µM oridonin group. **c**, **d** The mRNA levels of NQO1, HO-1 in MG-63 and HOS cells were measured by real-time PCR. The results are shown as means ± SD from three independent experiments. **P < 0.01 compared with control group; ^##^*P* < 0.01 compared with 15 µM oridonin group. **e** The level of intracellular ROS was measured by flow cytometry. The results are shown as means ± SD from three independent experiments. **P < 0.01 compared with control group; ^##^*P* < 0.01 compared with 15 µM oridonin group. **f** The respective apoptosis rates of different groups were measured by Annexin V/PI staining. The results are representative of at least three independent experiments and shown as mean ± SD. **P < 0.01 compared with control group; ^##^*P* < 0.01 compared with 15 µM oridonin group

We further used Nrf2 plasmid to detect whether the effect of oridonin on ROS generation was associated with Nrf2 signaling pathway in vitro. Western blot analysis showed that Nrf2 protein was overexpressed by Nrf2 plasmid (Figs. 5a, b).The inhibitory effect of oridonin on the protein expression and mRNA levels of NQO1 and HO-1 were reversed by Nrf2 plasmid transfection (Figs. 5a-d). Nrf2 plasmid transfection blocked oridonin-triggered generation of ROS (Fig. 5e). This suggested that oridonin triggered ROS generation by inhibiting Nrf2 in MG-63 and HOS cells. Moreover, Nrf2 plasmid attenuated oridonin-induced apoptosis in MG-63 and HOS cells (Fig. 5f). Collectively, these results indicated that oridonin induced apoptosis by triggering ROS generation via inhibiting Nrf2 signaling pathway in human osteosarcoma cells.

### Oridonin inhibited tumor growth by inducing apoptosis through activating PPAR-γ and inhibiting Nrf2 signaling pathway in vivo

**Fig. 6:**
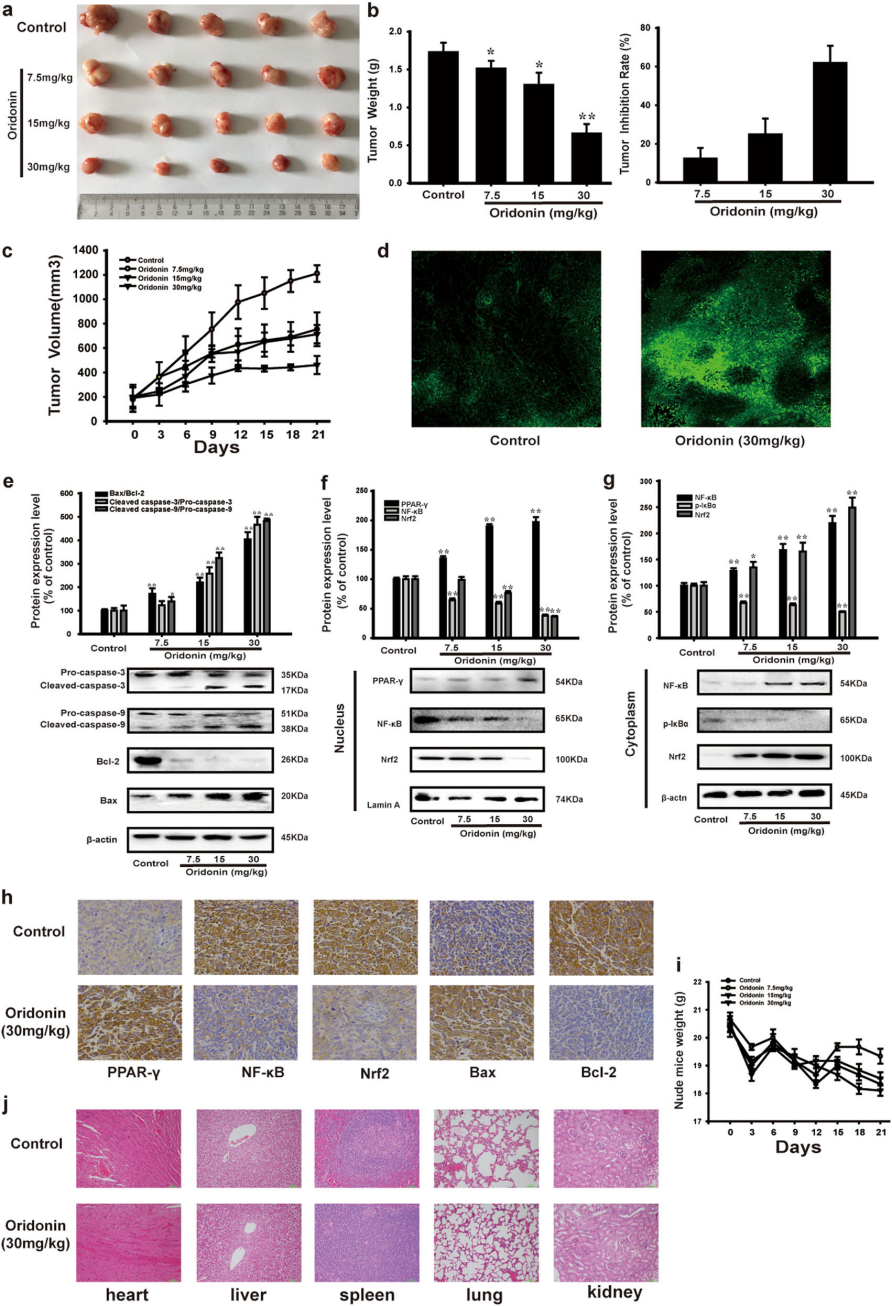
Oridonin inhibited tumor growth by inducing apoptosis through activating PPAR-γ and inhibiting Nrf2 signaling pathway in vivo. The nude mice bearing HOS osteosarcoma were treated with 30, 15, 7.5 mg/kg of oridonin by every 2 days. Control group was treated with normal saline. **a** The picture of nude mice xenograft tumors was captured after the oridonin treatment for 21 days. **b** Tumor mass were weighed, and tumor inhibition rate (%) = [(tumor weight of control – tumor weight of treated)/ tumor weight of control] × 100% was statistically analyzed. **c** Tumor volumes were measured and calculated every 3 days. **d** The DNA damage in tumor tissues was determined by TUNEL test (200 × ). **e** The protein expression of apoptosis-related proteins including Pro-caspase-3, Pro-caspase-9, Cleaved caspase-3, Cleaved caspase-9, Bcl-2 and Bax in tumor tissues were determined by Western blot analysis. β-actin was used as the loading control. Gray scale analysis was performed to determine the relative ratios of Bax/Bcl-2, Cleaved caspase-3/Pro-caspase-3, Cleaved caspase-9/Pro-caspase-9. The results are shown as means ± SD from three independent experiments. *P < 0.05, **P < 0.01 compared with control group. **f** The nuclear protein expression of PPAR-γ, NF-κB and Nrf2 in tumor tissues were determined by Western blot analysis. Lamin A was used as the loading control. Gray scale analysis was performed to determine the relative ratios of PPAR-γ, NF-κB and Nrf2. The results are shown as means ± SD from three independent experiments. **P < 0.01 compared with control group. **g** The cytoplasmic protein expression of NF-κB, p-IκBα and Nrf2 in tumor tissues were determined by Western blot analysis. β-actin was used as the loading control. Gray scale analysis was performed to determine the relative ratios of NF-κB, p-IκBα and Nrf2. The results are shown as means ± SD from three independent experiments. *P < 0.05, **P < 0.01 compared with control group. **h** The protein expression of PPAR-γ, NF-kB, Nrf2, Bax and Bcl-2 in nude mice xenograft tumors were detected by immunohistochemistry. The results are representative of at least three independent experiments. **i** Body weight was measured every 3 days. **j** The major organs (heart, liver, spleen, lung, and kidney) from treated and control mice was analyzed by H&E stained (200×)

To fully evaluate the anticancer effect of oridonin on osteosarcoma, we established a xenograft model in mice bearing HOS cells in vivo. There were significant differences in tumor volume between treatment groups and control group in the study. Oridonin markedly inhibited tumor growth and decreased the tumor volume (Figs. 6a-c). We used TUNEL staining assay to detect the effect of oridonin on the numbers of DNA damage of apoptotic cells in osteosarcoma tumor xenografts. The results depicted a dramatic increase in the TUNEL positive cells in the tumor tissues from oridonin-treated mice compared to the control group (Fig. 6d). Moreover, oridonin dose-dependently increased the ratio of Bax/Bcl-2 and the cleavage of Caspase-3 and Caspase-9 in tumor tissues (Fig. 6e). Oridonin also increased PPAR-γ expression and inhibited nuclear translocation of NF-κB and Nrf2 in the tumor tissues (Figs. 6f, g). These results were further confirmed by immunohistochemistry staining analysis (Fig. 6h). Our data demonstrated that oridonin inhibited tumor growth by inducing apoptosis through activating PPAR-γ and inhibiting Nrf2 signaling pathway in vivo.

Although oridonin inhibited the growth of osteosarcoma in vivo, its possible toxicity must be assessed comprehensively. For the duration of treatment (21 days), the body weight of mice between oridonin-treated and control groups was no significant difference (Fig. 6i). Moreover, no obvious change in major organs can be observed between control and oridonin-treated groups (Fig. 6j). Taken together, our study demonstrated that oridonin inhibited tumor growth with high safety by inducing apoptosis through activating PPAR-γ and inhibiting Nrf2 signaling pathway in human osteosarcoma.

## Discussion

Osteosarcoma, the prevailing malignant bone tumor, occurs mainly in children and adolescents. The prognosis of osteosarcoma has been improved dramatically in the last 30 years, however the 5-year survival rate is not remarkably increased to date^39,40^. The current drugs for osteosarcoma are limited for the severe toxicities, for instance, doxorubicin with well-documented cardiotoxicity^41,42^. Therefore, it has become a great deal of attention to search for novel more effective but less toxic chemopreventive and anticancer agents. In the current study, we investigated the anti-cancer effect of oridonin on osteosarcoma and unravelled the underlying mechanisms. Our findings demonstrated that oridonin induced apoptosis in osteosarcoma by activating PPAR-γ and inhibiting Nrf2 pathway in vitro and in vivo.

Apoptosis is a cellular procedure that is regulated by a variety of executioner and regulatory molecules^43^. The aberrant function of these regulators is extremely essential to the growth of different tumors and confers resistance to anticancer drugs. Both the inactivation of pro-apoptotic Bcl-2 family proteins and the activation of anti-apoptotic family members are important for the regulation of apoptotic dysregulation in cancer^44^. The anti-apoptotic Bcl-2 family proteins are widely over-expressed in cancer cells to overcome stress signals and thus related to the relaspse, poor prognosis, and resistance to chemotherapy^45^. Therefore, apoptosis has become one of the main molecular targets for drug discovery and development, especially for cancer. Our results revealed that oridonin induced mitochondrial apoptosis by increasing Bax/Bcl-2 ratio, loss of mitochondrial membranepotential, triggering ROS generation and inducing caspase-9 and caspase-3 cleavage in MG-63 and HOS cells. In vivo study also showed that oridonin induced mitochondrial apoptosis in tumor tissues of xenograft model. These findings suggested that oridonin induced mitochondrial apoptosis in osteosarcoma.

PPAR-γ can regulate cell differentiation, growth and apoptosis and is implicated in the several types of cancer pathogenesis and progression^46^. The effect of PPAR-γ ligands on cell growth inhibition and apoptosis has been investigated in various cancers^47^. Recent research has showed that knockdown of PPAR-γ expression reduces the cleavage of caspase 3 during the early stages of apoptosis^48^. Moreover, studies have documented that PPAR-γ activation might be a promising target for osteosarcoma. These properties make PPAR-γ activation by natural and synthetic ligands as an attractive strategy for cancer treatment and prevention. Therefore, agents that regulate PPAR-γ activation are being actively pursued. In our study, oridonin enhanced the protein expression of PPAR-γ in MG-63 and HOS cells. To confirm the effect of oridonin on apoptosis by PPAR-γ activation, we used GW9662, a specific inhibitor of PPAR-γ. The results showed that GW9662 reversed the pro-apoptotic effect of oridonin. It suggested that oridonin induced apoptosis by PPAR-γ activation.

Activating PPAR-γ displays the anticancer effects through inhibiting the activation of NF-κB^14,15^. The downstream target genes of NF-κB are implicated in the progression of cancer, such as apoptosis, proliferation and migration. Aberrant NF-κB activation has been observed in numerous human cancers^49–52^. Therefore, NF-κB has became an interesting therapeutic target for cancer therapy. Moreover, the studies have reported that inhibiting NF-κB activation causes apoptosis and cell cycle arrest in osteosarcoma cells as well^53,54^. In the present study we demonstrated that oridonin inhibited the nuclear translocation and activation of NF-κB in osteosarcoma. Our further results showed that GW9662 and PPAR-γ siRNA withdraw the inhibitory effect of oridonin on the nuclear translocation and activation of NF-κB. The pro-apoptotic effect of oridonin was also reversed by GW9662, indicating that oridonin induced apoptosis via inhibiting NF-κB activation by activating PPAR-γ. Our further in vivo results demonstrated that oridonin induced apoptosis in tumor tissues via inhibiting NF-κB activation by activating PPAR-γ.

ROS plays multiple roles in many types of cells, especially important in cell death and signaling^55^. Low levels of ROS act as second messengers in intracellular signaling and are necessary for normal cellular functions, whereas excessive ROS damage cell functions and promote cell death progession^56^. Cancer cells are susceptible to damage by exogenous drug-induced ROS, due to the imbalance of the redox homeostasis^57,58^. In our study, oridonin triggered high level generation of ROS in osteosarcoma cells MG-63 and HOS. It has been reported that ROS levels is mainly controlled by the transcription factor Nrf2^38^. Recent study has documented that Nrf2 was overexpressed in osteosarcoma tissues, and the 5-year survival rate was remarkably higher in patients with negative Nrf2 expression than in those with positive expression^27^. Therefore, inhibiting Nrf2 signaling pathway may be a potential target for the treatment of osteosarcoma. Our results showed that oridonin inhibited the nuclear translocation of Nrf2 and downregulated Nrf2 target genes NQO1 and HO-1 in the osteosarcoma cells. Oridonin suppressed the protein expression of NQO1 and HO-1 as well. The inhibitory effect of oridonin on NQO1 and HO-1 was reversed by Nrf2 plasmid transfection. Nrf2 plasmid transfection also blocked oridonin-triggered generation of ROS. Moreover, oridonin-induced apoptosis was remarkably withdrawn by Nrf2 plasmid transfection. These results showed that oridonin induced apoptosis by triggering ROS generation via inhibiting Nrf2 activation in osteosarcoma. Our in vivo study documented that oridonin inhibited Nrf2 activation in tumor tissues. Furethermore, the standard toxicology studies suggested that oridonin may exert antitumor activity with low levels of toxicity in vivo.

**Fig. 7:**
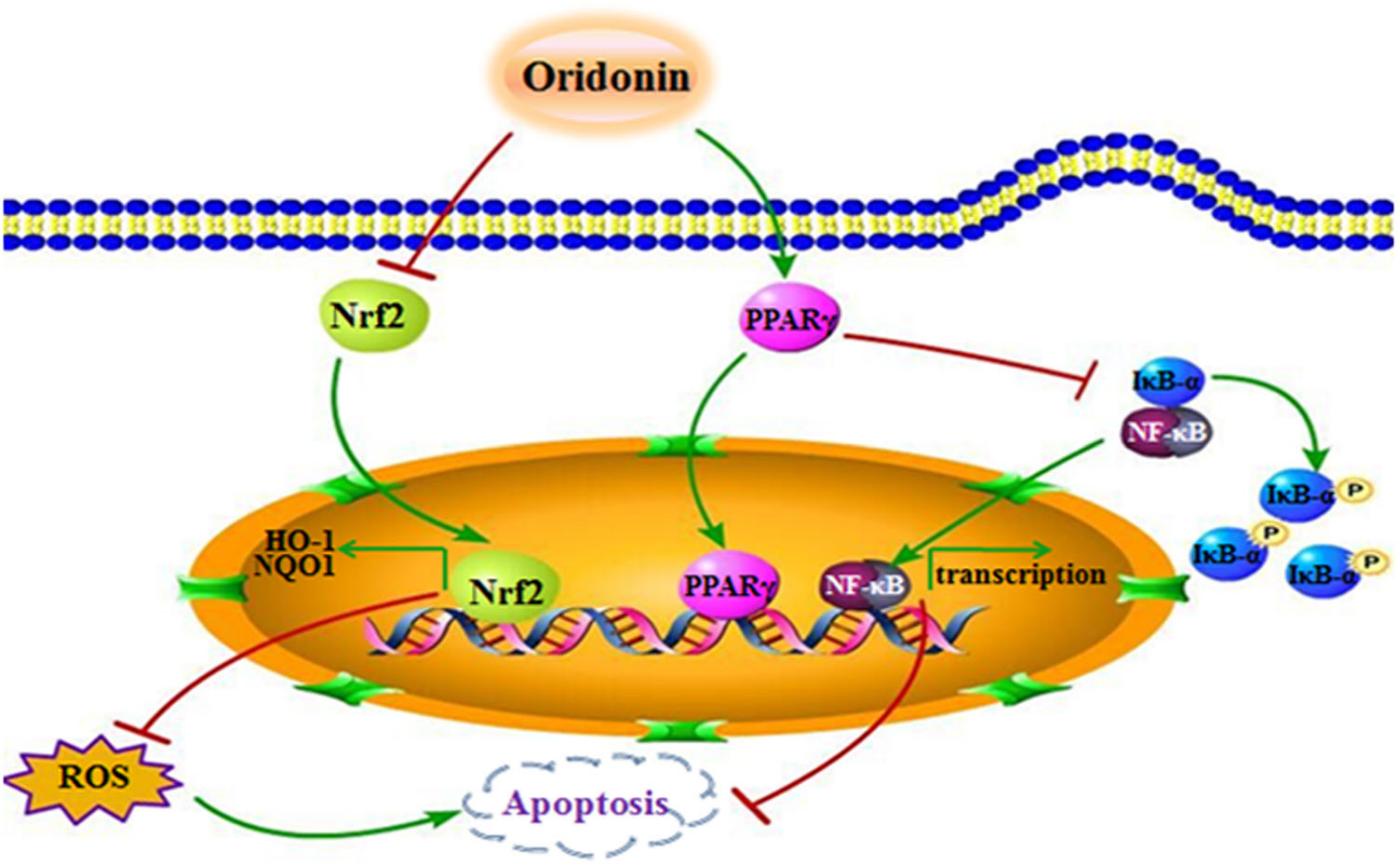
Possible mechanisms of oridonin-induced mitochondrial apoptosis in osteosarcoma

In summary, as a natural compound from Chinese herb medicine, oridonin induced apoptosis and inhibited tumor growth by activating PPAR-γ and inhibiting Nrf2 pathway in the osteosarcoma cells and xenograft tumor model (Fig. 7). Thus, these compelling evidences indicated that oridonin may be a potential and effective candidate against human osteosarcoma for its well anticancer efficiency and high safety.

## Materials and methods

### Reagents and cell culture

Oridonin with purity greater than 98%, were purchased from shanghai yuanye Bio-Technology Co., Ltd(Shanghai, China). Modified Eagle Medium (MEM), fetal bovine serum (FBS), benzyl penicillin and streptomycin were purchased from Gibco/BRL (Gaithersburg, MD, USA). 3-(4,5-dimethylthiazol-2-yl)-2,5-diphenyltetrazolium bromide (MTT) was obtained from solarbio (Beijing, China).

Primary antibodies against PPAR-γ, NF-κB were purchased from Santa Cruz Biotechnology (Santa Cruz, California). Antibodies against Nrf2, NQO1, HO-1, p-p65, p-IκBα, Caspase-3, Caspase-9, Bcl-2, Bax, β-actin, Lamin A were purchased from Cell Signaling Technology (Danvers, MA, USA). The human osteosarcoma cell lines MG-63, U-2OS, HOS and Saos-2, the human normal cells L02, HEK-293, HUVEC and hFOB 1.19, and human lung cancer cell line A549, human pancreatic carcinoma cell BxPC-3, human colorectal carcinoma cell line HCT-116, human cervical carcinoma cell line Hela, human ovarian cancer cell line SKOV-3, human gastric carcinoma cell line AGS, human leukemia cell line K562 were obtained from Cell Bank of Shanghai Institute of Biochemistry and Cell Biology, Chinese Academy of Sciences. The MG-63 and HOS cells were cultured in Minimum essential medium (Gibco; Thermo Fisher Scientific), while Saos-2, U-2OS and SKOV-3 cells were cultured in McCoy ‵5 A medium (Gibco; Thermo Fisher Scientific), supplemented with 10% FBS and 100 U/ml benzyl penicillin and 100 μg/ml streptomycin. The HEK-293, HUVEC, BxPC-3, HCT-116, Hela cells were cultured in Dulbecco’s minimum essential medium (Gibco; Thermo Fisher Scientific) with 10% FBS, and L02, A549, AGS, K562 was cultured in Roswell park memorial institute-1640 (Gibco; Thermo Fisher Scientific) with 10% FBS, with 100 units/ml penicillin and 100 μg/ml streptomycin. The cells were cultured at 37 °C, hFOB 1.19 were cultured at 33.5 °C, in 5% CO_2_.

### Cell morphological assessment

The MG-63 and HOS cells were plated in six-well plates and incubated overnight. After treatment with 0, 5, 10, and 15 µM oridonin for 24 h, cells were then incubated with 4,6-diamino-2-phenyl indole (DAPI) (Beyotime, China) in the dark for 10 min and washed with phosphate buffer saline (PBS) twice. The cells were observed under a fluorescence microscope (Olympus, Tokyo, Japan) to detect nuclei fragmentation and chromatin condensation.

### MTT assay

The MG-63 and HOS cells were cultured in 96-well plates and incubated overnight. After incubation with oridonin at various concentrations for 24 or 48 h, 20 μl MTT (5 mg/mL) was added to each well, and cells were incubated for another 4 h. The absorbance was measured at 490 nm with a MR7000 microplate reader (Dynatech, NV, USA). IC_50_ value was calculated by the software Graphpad Prism. All experiments were performed in triplicate in a parallel manner.

### Annexin V/PI staining

In brief, the MG-63 and HOS cells were seeded in six-well plates and treated with oridonin at concentrations of 5, 10,15 μM for 24 h, followed by washing with PBS twice and resuspended in PBS after oridonin exposure, then the cells were stained with the Annexin V/PI Cell Apoptosis Detection Kit (KeyGen, Biotech, Nanjing, China) according to the manufacturer’s protocol. The apoptosis rates of the cells were analyzed by flow cytometry (BD Biosciences, San Jose, CA, USA).

### Measurement of MMP

Quantitative changes of MMP was monitored using the MMP-specific fluorescent probe 5, 5′, 6, 6′-Tetrachloro-1, 1′, 3, 3′-tetraethyl-imidacarbocyanine iodide (JC-1) Apoptosis Detection Kit (KeyGen, Biotech, Nanjing, China). Briefly, oridonin-treated cells were washed with PBS twice, then incubated with JC-1 for 20 min in the dark at 37 °C. Then the stained cells were washed twice with cold buffer, resuspended and analyzed by a flow cytometer.

### Analysis of Caspase-3 and Caspase-9 activities

The Caspase-3 and Caspase-9 activities of MG-63 and HOS cells were measured using Caspase Activity Kit (KeyGen Biotech, Nanjing, China) according to the manufacturer’s protocol. Samples were measured with Relative Fluorescence Unit (RFU) at 405 nm.

### Measurement of ROS

Generation of intracellular ROS was detected using fluorescent dye 2,7-dichlorofluorescein-diacetate (DCFH-DA, Beyotime, China). The MG-63 and HOS cells were exposed to 5, 10,15 μM oridonin for 24 h respectively. The cells were collected and resuspended with DCFH-DA(10 μM), and incubated for 30 min in the dark at 37 °C. The level of ROS was determined by using fluorescence microscopy and flow cytometer.

### Quantitative real-time PCR analysis

Total RNA was isolated using the TriPure Solution (Takara Bio, Inc., Otsu, Shiga, Japan) after oridonin treatment, and then generate cDNA templates by reverse transcription reaction using Primescript reverse transcriptase (Takara Bio, Inc.) according to the manufacturer’s instructions. Then, the cDNAs were used as templates for determining the expression of related genes by quantitative real-time PCR. Each assay was done in triplicate.

### Western blot analysis

The MG-63 and HOS cells were seeded and incubated with various concentrations of oridonin for 24 h, and then cells were washed with cold PBS and lysed in 300 µl lysis buffer. Protein concentrations were quantified using the BCA assay method with Varioskan spectrofluorometer and spectrophotometer (Thermo, Waltham, MA) at 562 nm. Cell lysates were then subjected to SDS-PAGE and transferred onto polyvinylidene difluoride (PVDF) Immobilon-P membrane (Bio-Rad, USA) using a transblot apparatus (Bio-Rad, USA). The membranes were blocked with 5% skim milk powder at 37 °C for 1 h and incubated, each membrane was incubated with antibodies overnight at 4°C, and then the membranes were incubated with the secondary HRP-conjugated antibody (1:500) (Invitrogen, Carlsbad, USA) for 1 h at room temperature. Finally, the protein membranes were observed with an Odyssey Scanning System (Li-COR., Lincoln, Nebraska, USA).

### Immunofluorescence microscopy

The MG-63 cells were pretreated with oridinin (15 μM) for 24 h. Treated MG-63 cells were collected and cultured onto glass coverslips processed for immunofluorescence. Cells were then fixed, permeabilization and blocked before incubated with primary antibodies (1:50) against NF-κB and Nrf2 overnight at 4 °C. After washing with PBS, the cells were exposed to FITC-conjugated secondary antibodies (1:100). Then the coverslips were stained with DAPI for 30 min. The images were photographed with an Olympus FV1000 confocal microscope.

### Xenograft murine model of HOS cells

A 35–40 days old Male BALB/c nude mice, weighing 18–22 g, were supplied by Comparative Medicine Centre of Yangzhou University. The animal study was carried out according to National Institutes of Health regulations and approved by the Institutional Animal Care and Use Committee. The mice were maintained in a pathogen-free environment (21 ± 2 °C and 45 ± 10% humidity) on a 12 h light and 12 h dark cycle with food and water supplied freely during the entire experimental. On day 1, 5 × 10^6^ HOS cells suspended in 100 μl PBS were subcutaneously inoculated in the right flank of each nude mice. After 10–12 days, when tumor sizes reach around 80–150 mm^3^, then nude mice with similar tumor volume were randomly assigned to four groups (with 6 nude mice/group). Oridonin (7.5, 15, 30 mg/kg) groups received intraperitoneal injection of 7.5, 15, 30 mg/kg/2 days respectively. The control group was administered saline. Tumor volume (TV) was were measured daily to observe dynamic changes in tumor growth and calculated according to the formula: TV (mm^3^) = 0.5 × *d*^2^ × *D*, where *d* and *D* are the shortest and the longest diameters, respectively. After 21 days of oridonin administration, when the tumors of control group reached around 1400 mm^3^, all nude mice were sacrificed.

### TUNEL assay

The terminal deoxynucleotidyl transferase-mediated dUTP nick-end labeling (TUNEL) assay was used to analyze the apoptosis induction in the tissue specimen. It was carried out on Xenograft murine model treated as previously described using an in situ cell death detection kit following the manufacturer’s protocol. The slides were photographed under an Olympus FV1000 confocal microscope.

### Immunohistochemistry

The protein expression of PPAR-γ, NF-κB, Nrf2, Bax, Bcl-2 of the tumor tissues was assessed as described in the previous study^59^.

### Transfection of PPAR-γ siRNA, PPAR-γ plasmid and Nrf2 plasmid

The MG-63 and HOS cells were seeded in six-well plates followed by PPAR-γ siRNA, PPAR-γ plasmid and Nrf2 plasmid transfections according to the manufacturer’s protocol of Lipofectamine 2000 reagent (Invitrogen, Carlsbad, CA, USA) as described previously^59^. After transfections, the MG-63 and HOS cells were exposed to oridonin and harvested for further experiments.

### Statistical analysis

All data were shown as mean ± standard deviation from at least three independent experiments, each in triplicate samples for individual treatment or dosage. Statistical analyses were performed using ANOVA coupled with a post hoc test. All comparisons are made relative to untreated controls and significance of difference is indicated as *P < 0.05 and **P < 0.01.

## Electronic supplementary material

Supplement Figure 1

Supplement Figure 2

Supplement Figure 3

Supplement Figure Legends
