# Supplementary figures and images for: Oridonin exerts anticancer effect on osteosarcoma by activating PPAR-γ and inhibiting Nrf2 pathway
Source: Cell Death Dis. 2018 Jan 11;9(1):15. doi: 10.1038/s41419-017-0031-6 (PMC5849031; doi:10.1038/s41419-017-0031-6)

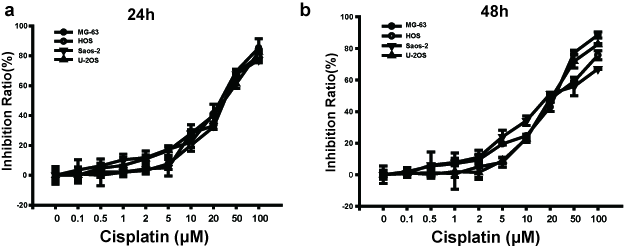

Supplement: Supplementary file 1 — Supplement Figure 1 [file 41419_2017_31_MOESM1_ESM.tif]

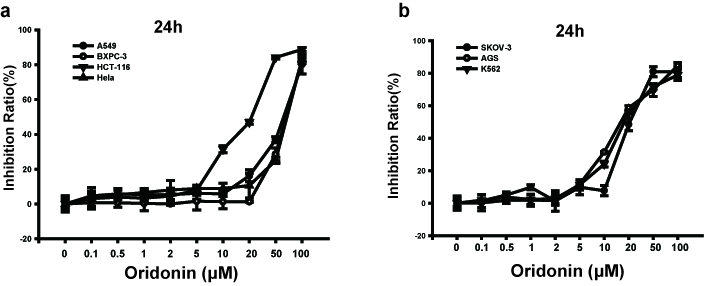

Supplement: Supplementary file 2 — Supplement Figure 2 [file 41419_2017_31_MOESM2_ESM.tif]

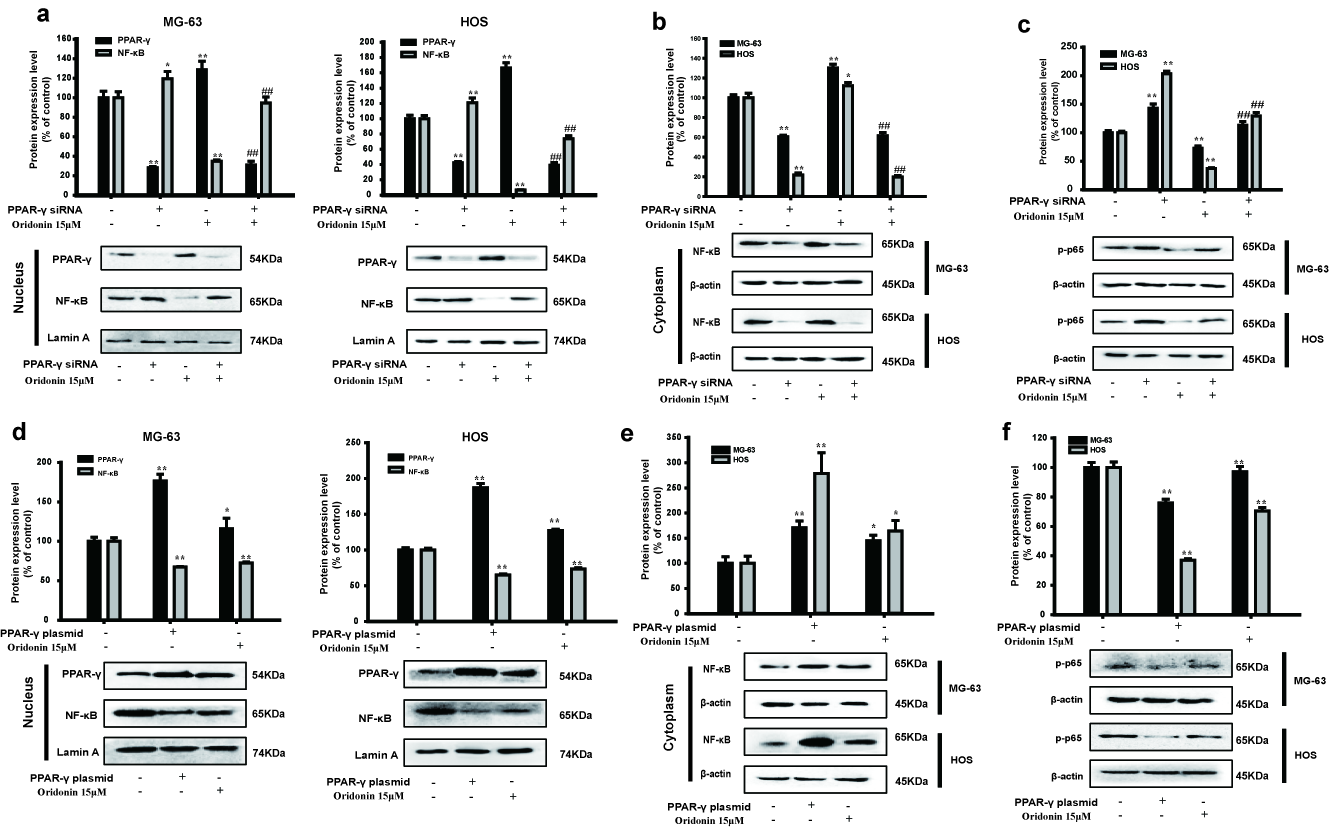

Supplement: Supplementary file 3 — Supplement Figure 3 [file 41419_2017_31_MOESM3_ESM.tif]
