# Supplementary material for: Oridonin exerts anticancer effect on osteosarcoma by activating PPAR-γ and inhibiting Nrf2 pathway
Source: Cell Death Dis. 2018 Jan 11;9(1):15. doi: 10.1038/s41419-017-0031-6 (PMC5849031; doi:10.1038/s41419-017-0031-6)
Supplement: Supplementary file 4 — Supplement Figure Legends [file 41419_2017_31_MOESM4_ESM.docx]

**Oridonin exerts anticancer effect on osteosarcoma by activating PPAR-γ and inhibiting Nrf2 pathway**

**Supplement Figure Legends**

**Supplement Figure 1** Oridonin inhibited the cell viability of various human cancer cells. (a) The effect of oridonin on the cell viability of A549, BXPC-3, HCT-116 and Hela cells after 24h treatment. (b) The effect of oridonin on the cell viability of SKOV-3, AGS and K562 cells after 24h treatment.

**Supplement** **Figure 2** Cispatin inhibited the cell viability of human osteosarcoma cells. (a) The effect of cisplatin on the cell viability of MG-63, HOS, Saos-2 and U-2OS cells after 24h treatment. (b) The effect of cisplatin on the cell viability of MG-63, HOS, Saos-2 and U-2OS cells after 48h treatment.

**Supplement Figure 3** Oridonin inhibited NF-κB signaling pathway via activating PPAR-γ in MG-63 and HOS cells. (a-c) MG-63 and HOS cells were incubated with oridonin (15 µM) for 24 h after transfection of PPAR-γ siRNA. (a) Nuclear protein expression of PPAR-γ and NF-κB and (b) Cytoplasmic protein expression of NF-κB were detected by Western blot. Lamin A and β-actin were used as nuclear and cytoplasmic markers, respectively. Gray scale was performed to determine the relative ratios of PPAR-γ, NF-κB. The results are shown as means±SD from three independent experiments. **P* < 0.05, ***P* < 0.01 compared with control group, ^##^*P* < 0.01 compared with 15 µM oridonin group. (c) The protein expression of p-p65 in MG-63 and HOS cells were determined by Western blot. β-actin was used as an internal control. Gray scale analysis was performed to determine the relative ratio of p-p65. The results are shown as means±SD from three independent experiments. ***P* < 0.01 compared with control group; ^##^ *P* < 0.01 compared with 15 µM oridonin group. (d-f) MG-63 and HOS cells were incubated with oridonin (15 µM) for 24 h or transfected with PPAR-γ plasmid. (d) Nuclear protein expression of PPAR-γ and NF-κB and (e) Cytoplasmic protein expression of NF-κB were detected by Western blot. Lamin A and β-actin were used as nuclear and cytoplasmic markers, respectively. Gray scale was performed to determine the relative ratios of PPAR-γ, NF-κB. The results are shown as means±SD from three independent experiments. **P* < 0.05, ***P* < 0.01 compared with control group. (f) The protein expression of p-p65 in MG-63 and HOS cells were determined by Western blot. β-actin was used as an internal control. Gray scale analysis was performed to determine the relative ratio of p-p65. The results are shown as means±SD from three independent experiments. ***P* < 0.01 compared with control group.
